# 2716. Epidemiology of invasive fungal infection in pediatric liver transplant recipients: A retrospective, single-center study, 2012-2022

**DOI:** 10.1093/ofid/ofad500.2327

**Published:** 2023-11-27

**Authors:** Ji Young Lee, Kyoung Ihn, Hong Koh, Ji-Man Kang, Myoung Soo Kim

**Affiliations:** Yonsei University College of Medicine, Seoul, Seoul-t'ukpyolsi, Republic of Korea; Severance Hospital, Yonsei University College of Medicine, Seoul, Seoul-t'ukpyolsi, Republic of Korea; Yonsei University College of Medicine, Seoul, Seoul-t'ukpyolsi, Republic of Korea; Severance Children’s Hospital, Yonsei University College of Medicine, Seoul, Seoul-t'ukpyolsi, Republic of Korea; Severance Hospital, Yonsei University College of Medicine, Seoul, Seoul-t'ukpyolsi, Republic of Korea

## Abstract

**Background:**

Studies on liver transplant (LT) recipients have reported an incidence of invasive fungal infection (IFI) ranging from 2.5 to 5%; however, antifungal prophylaxis has not yet been approved by the Ministry of Food and Drug Safety in Korea. We investigated the incidence of early-onset IFI and its characteristics in pediatric LT recipients in Korea, and also explored the risk factors for IFIs.

**Methods:**

This is a retrospective, single-center study on children (< 19 years of age) who have undergone LT from 2012 to 2022 at Severance Hospital, Korea. IFI was defined as the detection of fungus in sterile fluid, tissue, and/or blood with symptoms of a fungal infection. Superficial fungal infection and colonization on the skin or urine were excluded. The primary outcome is the occurrence of IFI within 90 days of LT. The logistic regression method was used to analyze the risk factors for IFI.

**Results:**

A total of 104 LT children were included, among whom 6 received multiple transplantations, resulting in 111 LT cases. 42% (n = 44) were male, and the median age at LT was 4.0 years of age (IQR 1.0–11.0). Two thirds of the patients received LT due to biliary atresia (n = 69, 66%), and the remaining were diagnosed with acute liver failure (n = 17, 16%), miscellaneous causes (n = 11, 11%), and so on (n = 7, 7%). Antifungal prophylaxis, except nystatin gargle, was done in 12.8% of recipients.

IFI was identified in 19 patients (18%), and due to five multiple IFI events per patient, a total of 23 IFI events were noted at a median time of 16.5 days after LT (IQR 10-38). 87% of the pathogens detected were *Candida* species (*C. albicans* 40%, *C. parapsilosis* 35%, *C. auris* 15%), and *Aspergillus fumigatus* (13%). Antifungal susceptibility patterns are described in Table 1.

The risk of IFI was significantly increased in those who underwent acute re-transplantation, postoperative intervention, or surgery due to bleeding, vascular, and bile duct complications (OR = 4.6; 95% CI, 1.5–13.6; P = 0.006) *8*) and renal replacement therapy (OR=3.2; 95% CI, 1.1-8.8; *P*=0.028).

**Table.**
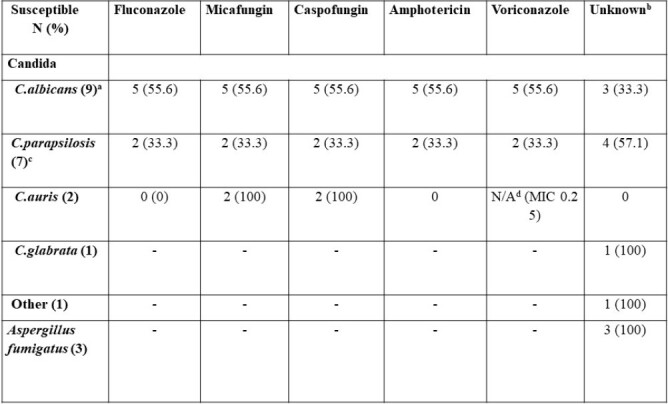
Antifungal susceptibility patterns of identified pathogens according to Clinical and Laboratory Standards Institute (CLSI). a Number of cases identified with the fungal pathogen b Antifungal susceptibility testing has to be additionally requested, so it was not performed in all cases. c 1 case had a fluconazole-resistant C.parapsilosis strain with intermediate resistance to other antifungal agents (MIC: fluconazole 32, micafungin 2, caspofungin 2, voriconazole 0.12, amphotericin B 0.5). d CLSI does not provide a tentative MIC breakpoint for voriconazole and other second generation azoles, but that of fluconazole may be considered as a surrogate.

**Conclusion:**

The incidence of early IFI after LT in Korean children is considerable; therefore, universal antifungal prophylaxis is necessary. High-risk IFI patients, such as those undergoing reoperation, intervention, or renal replacement therapy, require attention and close monitoring.

**Disclosures:**

**All Authors**: No reported disclosures

